# The prevalence and predictors of herb-drug interactions among Iranian cancer patients during chemotherapy courses

**DOI:** 10.1186/s12906-023-03869-1

**Published:** 2023-02-07

**Authors:** Maliheh Sadat Bazrafshani, Abbas Pardakhty, Behjat Kalantari Khandani, Haleh Tajadini, Sadra Ghazanfari Pour, Sara Hashemi, Shiva Amiri, Soheil Mehmandoost, Amin Beigzadeh, Samaneh Abbaszadeh, Hamid Sharifi

**Affiliations:** 1 HIV/STI Surveillance Research Center, and WHO Collaborating Center for HIV Surveillance, Institute for Futures Studies in Health, Kerman University of Medical Sciences, Kerman, 7616914111 Iran; 2 Pharmaceutics Research Center, Neuropharmacology Institute, Kerman University of Medical Sciences, Kerman, Iran; 3 Department of Internal Medicine, Hematology and Oncology Division, Kerman University of Medical Sciences, Kerman, Iran; 4 Physiology Research Center, Institute of Neuropharmacology, Kerman University of Medical Sciences, Kerman, Iran; 5 Student Research Committee, Kerman University of Medical Sciences, Kerman, Iran; 6 Departement of Medical Chemistry, Faculty of Pharmacy, Kerman University of Medical Sciences, Kerman, Iran; 7 Department of Toxicology and Pharmacology, Faculty of Pharmacy, Kerman University of Medical Sciences, Kerman, Iran; 8 Sirjan School of Medical Sciences, Sirjan, Iran

**Keywords:** Cancer, Chemotherapy, Herbal medicine, Food/Herbal drug interactions

## Abstract

**Background:**

The concurrent usage of herbal medicines with conventional therapies is an important concern in cancer treatment which can lead to unexpected consequences like herb-drug interactions. This study aimed to determine the prevalence of potential herb-drug interactions and to predict factors associated with herb-drug interactions for cancer patients.

**Methods:**

This cross-sectional study was conducted among a convenience sample of 315 cancer patients referring to the oncology clinics of Kerman city in 2018. Data were collected via comprehensive face-to-face interviews and medical chart reviews. A drug interaction checker was used to determine herb-drug interactions. The information of patients was compared based on herb-drug interactions using bivariable logistic regression models, and predictors were determined by the multivariable logistic regression model. All analyses were performed by Stata software version 16.

**Results:**

Of 262 patients (83.2% of the patients) who used herbal medicines, 209 patients [79.8% (95% Confidence Intervals (CI): 75.2 – 85.1)] had potential herb-drug interactions. Chamomile was the most popular herbal medicine (*n* = 163, 78.0%), and minor and moderate herb-drug interactions were caused by green tea (*n* = 34, 16.3%) and peppermint (*n* = 78, 37.5%). The number of chemotherapeutic agents (OR: 1.92, 95% CI: 1.43–2.58; *P*-value < 0.0001) and the experienced of pain during chemotherapy courses (OR = 2.22, 95%CI:1.00–4.94; *P*-value = 0.04) were some of the predictors of herb-drug interactions among cancer patients.

**Conclusion:**

Herbal medicine use during chemotherapy was found prevalent among cancer patients; of them, the experience of potential herb-drug interactions was highly frequent. Oncologists and clinical pharmacologists are recommended to take into account challenges associated with herb-drug interactions in their routine practices, particularly during chemotherapy among these patients.

**Supplementary Information:**

The online version contains supplementary material available at 10.1186/s12906-023-03869-1.

## Introduction

Cancer is one of the leading causes of death worldwide [1]. World Health Organization (WHO) reports that breast, colorectal, lung, and liver cancers had the highest incidence and mortality rate in the Eastern Mediterranean Region in 2020 [2]. These types of cancers with similar incidence and mortality are also observed in Iran, and there is an increment trend in the incidence and mortality rate for most cancers [3]. Surgery, radiation therapy, and systemic treatment are the main cancer treatment protocols applied separately or in combination for cancer patients [4]. Chemotherapy, as a systemic treatment of cancer, has many side effects, such as nausea and vomiting, diarrhea, mucositis, fatigue, and hair loss [5, 6].

To reduce the short-term and long-lasting side effects of chemotherapy, a great proportion of cancer patients use Complementary and Alternative Medicines (CAM) [7]; this usage is common without consultation provided by physicians and healthcare workers [8]. Among CAM therapies, herbal medicines are more popular among patients. Current reports show a remarkable and variable prevalence of using herbal medicine by cancer patients from 14% to 66.7% [9, 10], especially during conventional treatments or palliative care and chemotherapy (37% -38%) [11, 12]. In fact, concurrently using herbal medicines with conventional therapies is one of the most important concerns in cancer treatment which can lead to unexpected consequences [13].

In the pharmacokinetic interactions, herbal medicines, due to their pharmacokinetic properties, interact with chemical agents and affect the absorption, distribution, metabolism, and excretion of chemotherapeutic agents when orally used. The pharmacodynamics interactions are often lower clinically significant than pharmacokinetic interactions [14]. The herb-drug interactions in cancer patients are more important. However, the prevalence of this event among cancer patients is unknown, and limited studies have reported only a proportion of patients at risk for herb-drug interactions [13, 15]. Along with the unclear prevalence of herb-drug interactions in cancer patients, the epidemiological predictors of it are also unknown. Several studies have only reported the mechanisms of some herb-drug interactions [13, 16]. In this regard, a great proportion of Iranian cancer patients use herbal medicine in combination with a chemotherapeutic agent [17]. According to the lack of updated studies on this issue, this study aimed to determine the prevalence of potential herb-drug interactions among these patients and also identify the epidemiological predicting factors of herb-drug interactions for Iranian cancer patients.

## Material and methods

### subject and setting

The present cross-sectional study was conducted among 315 cancer patients referred to the oncology clinics of Kerman (one private and two governmental clinics) from February to June 2018. These patients were selected via a convenience sampling method, and sampling was not restricted to sex, cancer site, and clinical stage of cancer. The inclusion criteria were age above 18 years, and receiving at least one chemotherapy course through infusion, injection, or oral route. Patients who had completed the chemotherapy course one month before the survey were also eligible for the study.

### Data collection

Data were collected via comprehensive face-to-face interviews and also medical chart reviews. An interview form consisting of three parts was developed to conduct the reviews. In the first part, demographic variables like age, sex, marital status, place of residence, and educational level of patients were collected. The second part included the common name of herbal medicines that patients used in oral route during chemotherapy courses. In the last part of the form, common side effects of chemotherapy, such as constipation and diarrhea, nausea and vomiting, pain, and skin, or oral lesions, were asked. In this part, the status of other comorbidity illnesses was recorded. The first author interviewed all patients in waiting rooms of clinics before the start of the chemotherapy course or after its completion. Additional clinical information was obtained through a medical chart review. The cancer site, clinical stage, metastatic status, and recurrence status were in the clinical information category. To protect patients’ privacy, this part of the data collection was done by the staff of the oncology clinics.

### herb-drug interaction assessment

For checking herb-drug interactions, we used a drug interaction checker supported by natural medicine collaboration (URL: https://naturalmedicines.therapeuticresearch.com/). The scientific names of herbal medicines and the generic name of chemotherapeutic agents are checked together as a pair of herb-drug. In this part, two herbalists determined and approved the scientific name of herbal medicines. According to the interaction checker tool's results, which reports published evidence results, interactions were stratified into three levels: minor, moderate, and major based on the evidence (anecdotal evidence, theoretically based on pharmacology, in vitro studies, randomized and non-randomized clinical trials). If the risk of the adverse outcome appeared small, the potential minor drug interactions occurred. Moderate drug interactions may exacerbate the patient's disease and/or a change in the therapy. The severe drug interactions are life-threatening and/or require medical treatment or intervention to minimize or to prevent the severe adverse effects [18].

### Data analysis

Data were described using mean ± Standard Deviation (SD), frequency, percentage, and 95% Confidence Intervals (CIs). Demographic and clinical information of patients were compared based on herb-drug interactions by bivariable logistic regression models. Every variable with a *P*-value < 0.2 in bivariable models was selected and entered into the multivariable logistic regression model. The final model was fitted using backward elimination. In every step of multivariable models, variables with higher *P*-values were continuously removed from the model set until all remaining variables in the model were significant (*P*-value < 0.05). All descriptive and analytical analyses were performed by using Stata software version 16.

## Results

### Demographic and clinical information of cancer patients with herbal medicines

Of 315 cancer patients recruited for the study, 262 patients (83.2%; 95% CI: 78.6, 87.1) used at least one herbal medicine during chemotherapy courses and were included in this analysis. The mean ± SD age of cancer patients who used herbal medicines was 51.1 ± 14.0 years (age range: 18 to 92). More than 70% of the patients were females (*n* = 188, 71.8%) and urban residents (*n* = 204, 77.9%). The majority of cancer patients were married (*n* = 248, 94.7%). More than half of the patients were under diploma (*n* = 152, 58.0%). The breast was the most prevalent cancer site among patients (*n* = 98, 37.4%). Almost half of the patients had metastatic cancers (*n* = 117, 44.7%), but recurrence was not frequent among them (*n* = 45, 17.2%). More than half of the patients suffered from comorbidities (*n* = 138, 52.7%). Common complications among patients were nausea and vomiting (*n* = 186, 71.0%), constipation and diarrhea (*n* = 169, 64.5%), and pain (*n* = 160, 61.1%) (Table 1).

**Table 1:** Prevalence of herb-drug interactions based on demographic and clinical information of cancer patients referred to the outpatient clinics to receive chemotherapy courses (*n* = 262)

Variables	Level of variables	Cancer patients with herbal medicine	Prevalence of herb-drug interactions	95% CI
**Sex**	Male	74	51 (68.9)	58.1 – 78.4
	Female	188	158 (84.0)	78.7 – 89.3
**Marital status**	Single	14	11 (78.6)	57.1 – 100.0
	Married	248	198 (79.8)	74.6 – 84.7
**Area of resident**	Urban	204	164 (80.4)	75 – 85.3
	Rural	58	45 (77.6)	65.6 – 87.9
**Education level**	Under diploma	152	116 (76.3)	69.1 – 82.9
	Diploma and more	110	93 (84.5)	77.3 – 90.9
**Cancer site**	Breast	98	92 (93.9)	88.8 – 98
	Gastrointestinal	51	28 (54.9)	41.2 – 68.6
	Lymphoma and hematologic tumors	46	37 (80.4)	67.4 – 91.3
	Gynecologic	30	26 (86.7)	73.3 – 96.7
	Thorax	14	9 (64.3)	42.9 – 85.7
	Other sites	23	17 (73.9)	56.5 – 91.3
**Clinical stage**	I	18	12 (66.7)	44.4 – 88.9
	II	42	35 (83.3)	71.4 – 92.9
	III	48	39 (81.3)	70.8 – 91.7
	IV	37	33 (89.2)	78.4 – 97.3
	Unclear	117	90 (76.9)	68.4 – 84.6
**Metastatic status**	Negative	139	109 (78.4)	71.2 – 84.9
	Positive	117	96 (82.1)	75.2 – 88.9
	Unclear	6	4 (66.7)	33.3—100
**Recurrence status**	Negative	210	167 (79.5)	73.8 – 84.8
	Positive	45	38 (84.4)	73.3 – 93.3
	Unclear	7	4 (57.1)	28.6 – 85.7
**Comorbid illness**	Negative	124	96 (77.4)	70.2 – 84.7
	Positive	138	113 (81.9)	76.1 – 87.7
**Nausea and vomiting**	Negative	76	56 (73.7)	64.5 – 82.9
	Positive	186	153 (82.3)	76.9 – 87.1
**Constipation and diarrhea**	Negative	93	74 (79.6)	71 – 88.2
	Positive	169	135 (79.9)	73.4 – 85.8
**Pain**	Negative	102	70 (68.6)	59.8 – 77.5
	Positive	160	139 (86.9)	81.9 – 91.9
**Oral lesions**	Negative	134	102 (76.1)	68.7 – 83.6
	Positive	128	107 (83.6)	77.3 – 89.8
**Skin lesions**	Negative	130	100 (76.9)	70 – 84.6
	Positive	132	109 (82.6)	76.5 – 88.6

*CI* Confidence Intervals

### Prevalence of herb-drug interactions

Of 262 patients with herbal medicine, 209 patients [79.8% (95% CI: 75.2–85.1)] had potential herb-drug interaction. The prevalence of herb-drug interactions based on demographic and clinical information is shown in Table 1.

### Properties of chemotherapy regimens of cancer patients with interaction

**Fig. 1:**
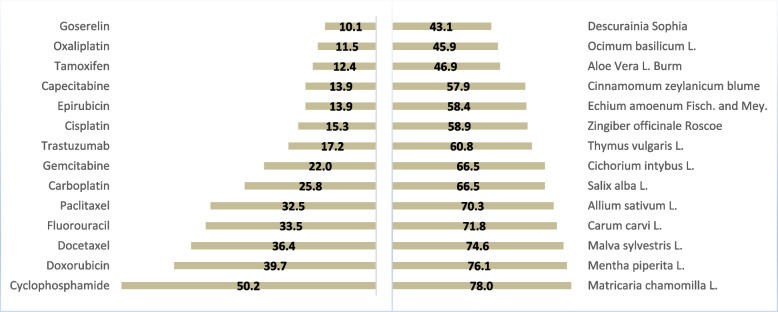
The common prescribed chemotherapeutic agents and popular herbal medicines among cancer patients with herb-drug interaction in oncology outpatient clinics, Kerman (*n* = 209) (Right: herbal medicines; Left: chemotherapeutic agents)

Thirty-three different chemotherapeutic agents were prescribed for cancer patients. The mean ± SD of prescribed medications was 4.0 ± 1.8 per patient (range: 1 to 10). Cyclophosphamide was the most frequent chemotherapeutic agent (*n* = 105, 50.2%). Arsenic Trioxide, Flutamide, Nivolumab, and Thalidomide (*n* = 1, 0.48%) were the least prescribed chemotherapeutic agents (Fig. 1).

### Properties of herbal medicines used by cancer patients with potential interaction

Cancer patients with potential herb-drug interaction reported 78 different herbal medicines used during chemotherapy courses. The mean ± SD number of herbal medicines among these patients was 12.6 ± 5.5 per patient (range: 2 to 29). *Matricaria chamomilla L.* (chamomile) was the most popular herbal medicine among cancer patients (Fig. 1).

### Frequency and types of herb-drug interactions

Based on the findings, 128 pairs of herbs and drugs can lead to potential herb-drug interactions. Of them, 19 and 116 pairs of herbs and drugs caused potential minor and moderate herb-drug interactions, respectively. No major herb-drug interaction was found. Potential moderate herb-drug interactions occurred in all patients (*n* = 209, 100%), while potential minor herb-drug interactions happened in less than a third of the patients (*n* = 67, 32.1%). The frequent potential minor and moderate herb-drug interactions were caused by *Camellia sinensis L****.*** (green tea) (*n* = 34, 16.3%) and *Mentha piperita L*. (peppermint) (*n* = 78, 37.5%), respectively, when used in combination with Cyclophosphamide. Among herbal medicines, *Camellia sinensis L.* (green tea), *Matricaria chamomilla L.* (chamomile), *Curcuma longa L.* (Turmeric), and *Silybum marianum L.Gaertn* (milk thistle) caused both potential minor and moderate herb-drug interactions. Potential minor and moderate herb-drug interactions are detailed in Additional file 1: Appendix A.

### Frequency of confirmed evidence for herb-drug interactions

**Fig. 2:**
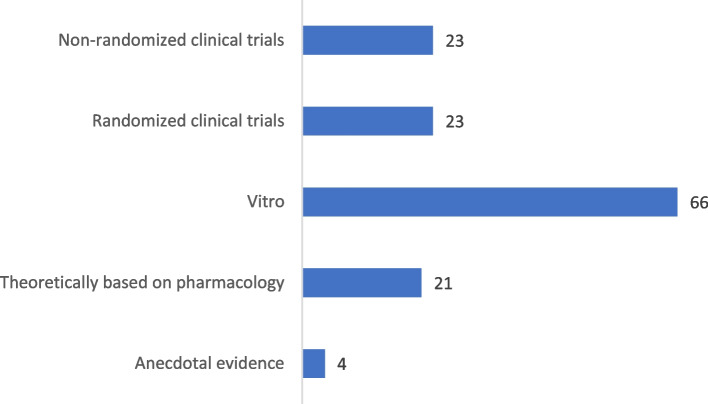
The frequency of confirmed evidence of herb-drug interactions among cancer patients in the oncology outpatient clinics, Kerman (*n* = 209)

A considerable number of herb-drug interactions was determined or confirmed by in vitro (66 studies) or clinical studies (46 studies, randomized and non-randomized clinical trials), and only four herb-drug interactions were based on anecdotal evidence (Fig. 2). All herb-drug interactions were confirmed by evidence except nine herb-drug interactions, which were confirmed by more evidence. These pairs were “Cyclophosphamide and *Mentha piperita L*.” (Vitro and randomized clinical trial), “Cyclophosphamide and *Glycyrrhiza glabra L.”* (Vitro and randomized clinical trial), “Docetaxel and *Glycyrrhiza glabra L.”* (Vitro and randomized clinical trial), “Doxorubicin and *Valeriana officinalis L.”* (Vitro and non-randomized clinical trial), “Paclitaxel and *Glycyrrhiza glabra L.”* (Vitro and randomized clinical trial), “Tamoxifen and *Mentha piperita L*.” (Vitro and randomized clinical trial), “Tamoxifen and *Foeniculum vulgare Mill”* (Vitro and theoretically based on pharmacology), “Tamoxifen and *Valeriana officinalis L.”* (Vitro and non-randomized clinical trial) and “Tamoxifen and *Glycyrrhiza glabra L.”* (Vitro and randomized clinical trial). The type of evidence for other herb-drug interactions is presented in Additional file 1: Appendix A.

### Mechanisms of actions of herb-drug interactions

Based on the reported evidence, in most drug interactions, induction or inhibition of hepatic and intestinal Cytochrome P450 3A4 (CYP3A4) occurred (139 cases; 69.8%). The first phase of metabolism of chemotherapeutic agents, which mostly depend on CYP3A4, was impaired. Other frequent mechanisms of herb-drug interactions were the dip in GI transit time (11 cases) and the plunge in the hepatotoxicity of chemotherapeutic agents (8 cases) (Table 2).

**Table 2:** Frequency of action mechanisms of herb-drug interactions among cancer patients referred to outpatient clinics to receive chemotherapy courses (*n* = 209)

Mechanisms of action	Frequency
Inhibit CYP3A4^a^	78
Induce hepatic CYP3A4	13
Induce intestinal CYP3A4	13
Inhibit hepatic CYP3A4	13
Inhibit intestinal CYP3A4	13
Decrease gastrointestinal transit time	11
Induce CYP3A4	9
Increase hepatotoxicity of the drug	8
Inhibit P-gp^b^	6
Inhibit OATP1A2 ^c^	4
Inhibit OATP1B1 ^d^	4
Inhibit OATP2B1 ^e^	4
Stimulate or suppress the immune function	4
Inhibit CYP2C9 ^f^	3
Inhibit CYP2C19 ^g^	2
Potential estrogenic effects of herbs	2
Inhibit UGT2B7 ^h^	2
Inhibit UGT1A1 ^i^	2
increase photosensitivity of drug	2
Inhibit or induce CYP2D6 ^j^	2
Enhances the oral bioavailability of drug	1
Inhibit CYP1A2 ^k^	1
Inhibit CYP2C8 ^l^	1
Block proteasome inhibitor	1

^a^Cytochrome P450 3A4

^b^P-glycoprotein

^c^Organic Anion-Transporting Polypeptide 1A2

^d^Organic Anion-Transporting Polypeptide 1B1

^e^Organic Anion-Transporting Polypeptide 2B1

^f^Cytochrome P450 2C9

^g^Cytochrome P450 2C19

^h^UDP-Glucuronosyl Transferase 2B7

^i^UDP-Glucuronosyl Transferase 1A1

^j^Cytochrome P450 2D6

^k^Cytochrome P450 1A2

^l^Cytochrome P450 2C8

### Demographic and clinical correlates of herb-drug interaction among cancer patients

The bivariable logistic regression models showed that herb-drug interactions were related to sex, cancer site, recurrence status, pain experienced as a chemotherapy complication, and the number of chemotherapeutic agents and herbal medicines. Based on the results, females (OR: 2.28, 95% CI: 1.22–4.26; *P*-value = 0.01) and patients with pain experienced during chemotherapy (OR: 3.16, 95% CI: 1.70–5.87; *P*-value < 0.0001) had greater odds of herb-drug interactions. By increasing the number of chemotherapeutic agents (OR: 1.75, 95% CI: 1.40–2.19; *P*-value < 0.0001) and herbal medicines (OR: 1.12, 95% CI: 1.05–1.20, *P*-value < 0.0001) the odds of herb-drug interactions increased. Patients with gastrointestinal cancers (OR: 0.09, 95% CI: 0.03–0.24, *P*-value < 0.0001) and other types of cancers (OR: 0.20, 95% CI: 0.04–0.95, *P*-value = 0.04) versus patients with breast cancer had a lower odd of herb-drug interactions. Also, patients with unclear recurrence status had lower odds of herb-drug interaction compared to patients with negative recurrence status (OR: 0.19, 95% CI: 0.04–0.89, *P*-value: 0.03) (Table 3).

**Table 3:** The bivariate logistic regression for comparing demographic and clinical information of cancer patients

Variable	Level of variable	cOR^a^ (95% CI^b^)	*P*-value
**Age**^**c**^		0.99 (0.97–1.01)	0.80
**Sex**	Male	1	–
	Female	2.28 (1.22–4.26)	0.01
**Marital status**	Single	1	–
	Married	1.05 (0.28–3.91)	0.93
**Area of residence**	Urban	1	–
	Rural	0.87 (0.42–1.76)	0.70
**Education level**	Under diploma	1	–
	Diploma and more	1.58 (0.84–2.97)	0.15
**Cancer site**	Breast	1	–
	Gastrointestinal	0.09 (0.03–0.24)	≤ 0.0001
	Lymphoma and hematologic tumors	0.31 (0.10–0.91)	0.03
	Gynecologic	0.69 (0.07–6.27)	0.74
	Thorax	0.33 (0.10–1.07)	0.06
	Other sites	0.20 (0.04–0.95)	0.04
**Clinical stage**	I	1	–
	II	2.5 (0.70–8.92)	0.15
	III	2.16 (0.64–7.33)	0.21
	IV	4.12 (0.98–17.19)	0.05
	Unclear	1.58 (0.54–4.62)	0.39
**Metastatic status**	Negative	1	–
	Positive	1.25 (0.67–2.34)	0.46
	Unclear	0.27 (0.05–1.43)	0.12
**Recurrence status**	Negative	1	–
	Positive	1.39 (0.58–3.34)	0.45
	Unclear	0.19 (0.04–0.89)	0.03
**Comorbid illness**	Negative	1	–
	Positive	1.37 (0.75–2.51)	0.29
**Nausea and vomiting**	Negative	1	–
	Positive	1.77 (0.94–3.31)	0.07
**Constipation and diarrhea**	Negative	1	–
	Positive	1.08 (0.58–2.02)	0.79
**Pain**	Negative	1	–
	Positive	3.16 (1.70–5.87)	≤ 0.0001
**Oral lesions**	Negative	1	–
	Positive	1.66 (0.90–3.06)	0.10
**Skin lesions**	Negative	1	–
	Positive	1.35 (0.73–2.46)	0.32
**Number of chemotherapeutic agents**^**c**^		1.75 (1.40–2.19)	≤ 0.0001
**Number of herbal medicines**^**c**^		1.12 (1.05–1.20)	≤ 0.0001

^a^Crude Odds Ratio

^b^Confidence Intervals

^c^These variables were considered as continuous in the analysis

### Predictors of herb-drug interactions

According to the results of the multivariable logistic regression model, the number of chemotherapeutic agents (OR: 1.92, 95% CI: 1.43–2.58; *P*-value < 0.0001), number of herbal medicines (OR: 1.15, 95% CI: 1.06–1.24, *P*-value < 0.0001), gastrointestinal cancers (OR: 0.08, 95% CI: 0.02–0.30, *P*-value < 0.0001), thorax cancers (OR: 0.10, 95% CI: 0.01–0.61, *P*-value = 0.01), stage IV cancer (OR: 8.42, 95% CI: 1.10–64.04, *P*-value = 0.04), unclear recurrence status (OR: 0.06, 95% CI: 0.005–0.67, *P*-value = 0.02) and the experience of pain during chemotherapy (OR = 2.22, 95%CI:1.00–4.94; *P*-value = 0.04) were determined as the predictors of herb-drug interactions among cancer patients (Table 4).

**Table 4:** The predicting factors for potential herb-drug interaction among cancer patients

Variable	Level of variable	AOR^a^ (95% CI^b^)	*P*-value
**Number of chemotherapeutic agents**^**c**^		1.92 (1.43–2.58)	< 0.0001
**Number of herbal medicines**^**c**^		1.15 (1.06–1.24)	< 0.0001
**Cancer site**	Breast	1	–
	Gastrointestinal	0.08 (0.02–0.30)	< 0.0001
	Lymphoma and hematologic tumors	0.41 (0.08–1.92)	0.26
	Gynecologic	0.28 (0.06–1.32)	0.27
	Thorax	0.10 (0.01–0.61)	0.01
	Other sites	0.58 (0.09–3.55)	0.56
**Clinical stage**	I	1	–
	II	2.66 (0.45–15.52)	0.27
	III	3.42 (0.59–19.74)	0.16
	IV	8.42 (1.10–64.04)	0.04
	Unclear	4.83 (0.93–25.03)	0.06
**Recurrence status**	Negative	1	–
	Positive	0.40 (0.11–1.36)	0.14
	Unclear	0.06 (0.005–0.67)	0.02
**Pain**	Negative	1	–
	Positive	2.22 (1.00–4.94)	0.04

^a^Adjusted Odds Ratio

^b^Confidence Intervals

^c^These variables were considered as continuous in the analysis

## Discussion

We found that more than eight out of ten cancer patients used herbal medicines during chemotherapy courses, and around eight patients out of ten with a history of herbal medicine consumption had potential herb-drug interactions. Potential moderate herb-drug interactions occurred in all patients, while potential minor herb-drug interactions happened in a third of patients. Chamomile was the most popular herbal medicine, and green tea leads to frequent potential minor and moderate herb-drug interactions. The number of chemotherapeutic agents, the number of herbal medicines, gastrointestinal cancers, thorax cancers, stage IV cancer, unclear recurrence status, and the experience of pain during chemotherapy courses were determined as the predictors of herb-drug interactions among cancer patients.

The high prevalence of using herbal medicines in combination with conventional treatments is an important issue addressed in many studies with different populations [19–22]. This finding has also been discussed in our study. Most cancer patients used herbal medicines during chemotherapy courses, and according to our previous study, this consumption was also hidden from physicians’ view [17]. Regardless of current treatments, patients use herbal medicines for various reasons. For example, patients believe they can use herbal medicines without trouble because they are natural, effective in treating diseases, reduce cancer symptoms, and have no side effects [23, 24]. These patients have mistaken beliefs about herbal medicines because herbs, when used in combination with drugs, influence the induction and inhibition of metabolic enzymes and, finally, on drug absorption [25]. Herb-drug interaction is the consequence of this combination and may lead to unexpected adverse clinical outcomes such as hepatotoxicity [26]. Studies showed that the prevalence of herb-drug interactions among cancer patients is considerable, and it varies from 2.3% [27] to 46% [28]. The findings of our study showed that more than three-quarters of cancer patients had herb-drug interactions, and this prevalence was higher in comparison to other studies. Some of the reasons for this discrepancy were related to more consumption of herbal medicines by our patients, consumption of herbal medicines which lead to herb-drug interactions such as garlic, green tea [29], and chamomile [30] and identification of new pairs of herb and drugs which result in interactions over time.

The results of our study are consistent with similar related studies [31, 32], indicating that most of the herb-drug interactions are caused by inhibition or induction of the CYP3A4 enzyme. As regards the metabolization of many drugs depending on this enzyme, induction or inhibition of it can lead to unexpected toxicity and under-treatment of cancer. In this regard, physicians and other healthcare workers must pay more attention and better prevent patients from using herbal medicines in combination with other drugs.

We also found that the number of herbal medicines and also chemotherapeutic agents as predictors increased the odds of herb-drug interactions among the patients. This result is in line with the study conducted by Levy et al. (2017) on hospitalized patients [33] and the study by Chi et al. (2020) on community-dwelling older adults [34]. The reason for this event is clear, and by increasing the number of each drug (herbal or chemotherapy), the odds of herb-drug interaction subsequently increased. Another predicting factor was experiencing pain during chemotherapy. A related study shows that a considerable proportion of patients with chronic pain used CAM [35]. Licorice [36], chamomile [37], and peppermint [38] as analgesics are popular herbal medicines and interact with chemotherapeutic agents. As a result, the experience of pain causes the use of herbal medicines, and the herb-drug interaction is the potential outcome of this usage.

Type of cancer and advanced cancer were other predictors of herb-drug interactions. Patients with gastrointestinal and thorax cancer had lower odds of herb-drug interactions versus patients with breast cancer. Two reasons can justify this difference. First, most patients with breast cancer are females, and females are more likely to use herbal medicines in combination with other drugs [39]. The second reason was related to the type of drugs used for patients with breast cancer. Tamoxifen, Letrozole, and Exemestane are common drugs that are usually prescribed for postmenopausal breast cancer patients. These patients used specific herbal medicines to reduce complications of menopause, such as licorice, fennel, and valerian, a supplement with an estrogenic activity that interacts with chemotherapeutic agents [40].

In contrast, patients with advanced cancers had greater odds of herb-drug interaction versus other patients. The results of one study showed that patients with advanced cancer were inclined to use CAM and the prevalence of using herbal medicines among these patients was considerable [41]. There are several reasons for these patients to use herbal medicines. They look for a way to reduce severe cancer symptoms and the side effects of chemotherapy courses. Also, they hope to live longer.

### Limitations

This study had four limitations. First, because some of the herbal medicines with one common name have several scientific names, we have restrictions on finding the scientific name of the herbal medicines. For this issue, the herbalists selected the prevalent species of herbal medicines in our country (Iran). Second, some patients may not remember the herbal medicines used due to recall bias. In this regard, we underestimated the prevalence of using herbal medicines in general and in each specific herbal medicine and the potential interactions. Third, we could not determine the exact time for using herbal medicine. So some of the used herbal medicines were not during the chemotherapy time. But based on the experience of cancer therapists in the region, patients prefer to use herbal medicine during chemotherapy to reduce the side effects of medicines. Fourth, as this was a cross-sectional study, we could not confirm the causation of the predictors with the potential herb-drug interaction as the outcome.

## Conclusion

Herbal medicine use during chemotherapy was found prevalent among cancer patients (more than eight out of ten patients). Among those with a history of herbal medicine use, the experience of potential herb-drug interactions was highly frequent (around eight out of ten). Some patients were at higher risk of the interaction including, patients with breast cancer, end-stage patients, and those with experienced pain during the treatment course. Oncologists and clinical pharmacologists are recommended to take into account challenges associated with herb-drug interactions in their routine practices, particularly during chemotherapy among these patients. Also, it is necessary to reduce the risk of herb-drug interactions with proper patient education, training their families, and consulting patients to prevent them from self-medication with herbal medicines by involving traditional medicine specialists in the treatment process of cancer patients. Extensive studies are recommended to determine the interactions between herbal medicines and chemotherapeutic agents.

## Supplementary Information

**Additional file 1.**

## Data Availability

The datasets generated and analyzed during the current study are not publicly available due to confidentiality but are available from the corresponding author at a reasonable request.
