# Supplementary material for: The prevalence and predictors of herb-drug interactions among Iranian cancer patients during chemotherapy courses
Source: BMC Complement Med Ther. 2023 Feb 7;23:41. doi: 10.1186/s12906-023-03869-1 (PMC9903537; doi:10.1186/s12906-023-03869-1)
Supplement: Supplementary file 1 — Additional file 1. [file 12906_2023_3869_MOESM1_ESM.docx]

Appendix A

| Herbal medicines | | Chemotherapeutic agents (Number of patients) | Type of evidence | Severity of interaction | Mechanism of action |
| --- | --- | --- | --- | --- | --- |
| Scientific name | Common name |  |  |  |  |
| ***Matricaria chamomilla L.*** | Chamomile | Cyclophosphamide (77), Docetaxel (59), Doxorubicin (58), Etoposide (9), Exemestane (16), Flutamide* (1), Imatinib (1), Letrozole (6), Paclitaxel (56), Tamoxifen (22), Vinblastine (5), Vincristine (12), Vinorelbine (9), Ifosfamide (10) | Vitro | Moderate | Inhibit CYP3A4, Inhibit CYP2C9, Inhibit CYP2D6 |
|  |  | Flutamide |  | Minor | Inhibit CYP1A2 |
| ***Mentha spicata L***. | Spearmint | Methotrexate (4), Bortezomib (7) | Theoretically based on pharmacology | Moderate | Increase marker of liver damage such as AST and ALT |
| ***Mentha piperita L***. | Peppermint | Cyclophosphamide (78), Docetaxel (56), Doxorubicin (60), Etoposide (10), Exemestane (13), Ifosfamide (8), Imatinib (2), Letrozole (5), Paclitaxel (53), Tamoxifen (19), Vinblastine (4), Vincristine (12), Vinorelbine (4) |  | Moderate | Inhibit CYP3A4,  Inhibit CYP2C19,  Inhibit CYP2C9, |
| ***Allium sativum L.*** | Garlic | Cyclophosphamide (75), Docetaxel (57), Doxorubicin (54), Exemestane (11), Ifosfamide (6), Imatinib (1), Letrozole (4), Paclitaxel (54), Tamoxifen (20), Vinblastine (2), Vincristine (12), Vinorelbine (7), Etoposide (10) | Non-randomized clinical trials | Moderate | Induce intestinal CYP3A4,  Inhibit hepatic CYP3A4 |
| ***Aloe Vera L. Burm*** | Aloe | Capecitabine (19), Chlorambucil (1), Cyclophosphamide (49), Exemestane (8), Flutamide (1), Imatinib (2), Letrozole (3), Methotrexate (3), Tamoxifen (13), Temozolomide (2), Topotecan (1), | Theoretically based on pharmacology | Moderate | Decrease GI transit time |
| ***Coriandrum sativum L.*** | Coriander | Fluorouracil (2) | Anecdotal evidence | Moderate | Increase photosensitivity of drug |
| ***Camellia sinensis L.*** | Green Tea | Bortezomib (2), Cyclophosphamide* (34), Etoposide* (4), Irinotecan (9), Methotrexate (2), Paclitaxel* (22), Tamoxifen (3) | Anecdotal evidence | Moderate | blocking proteasome inhibitory action,  Increase hepatotoxicity of the drug,  Inhibit OATP1A2, OATP1B1, and OATP2B1 |
|  |  | Cyclophosphamide, Docetaxel (27), Doxorubicin (25), Etoposide, Exemestane (6), Ifosfamide (4), Imatinib (1), Letrozole (2), Paclitaxel, Vinblastine (2), Vincristine (5), Vinorelbine (2) | Vitro | Minor | Increase hepatotoxicity of the drug,  Inhibit intestinal CYP3A,  Induce hepatic CYP3A4 |
| ***Nigella sativa L.*** | Black seed | Cisplatin (16), Cyclophosphamide (28), Thalidomide (1), | Vitro | Moderate | Stimulate or suppress the immune function |
| ***Foeniculum vulgare Mill*** | Fennel | Cyclophosphamide (24), Docetaxel (25), Doxorubicin (25), Etoposide (3), Exemestane (4), Ifosfamide (3), Letrozole (3), Paclitaxel (23), Tamoxifen (5), Vincristine (7), Vinorelbine (3) | Vitro and Theoretically based on pharmacology | Moderate | Inhibit CYP3A4, Decrease the antiestrogenic effect of tamoxifen, due to fennel's potential estrogenic effects. |
| ***Valeriana officinalis L.*** | Valerian | Cyclophosphamide (15), Docetaxel (9), Doxorubicin (9), Exemestane (4), Ifosfamide (1), Letrozole (2), Paclitaxel (10), Tamoxifen (4),  Vincristine (1), Vinorelbine (3) | Vitro and non-randomized clinical trials | Moderate | Inhibit CYP3A4,  Inhibit UGT1A1 and UGT2B7,  inhibit or induce CYP2D6 |
| ***Glycyrrhiza glabra L.*** | Licorice | Cyclophosphamide (5), Docetaxel (4), Doxorubicin (3), Exemestane (1), Letrozole (5), Paclitaxel (5), Tamoxifen (1), Vinblastine (1), Vincristine (1), Vinorelbine (1) | Vitro and randomized clinical trials | Moderate | Induce CYP3A4,  Inhibit CYP3A4,  Inhibit CYP2C19,  Inhibit CYP2C8,  Inhibit CYP2C9 |
| ***Berberis vulgaris L.*** | Berberis | Cyclophosphamide (3), Doxorubicin (1), Paclitaxel (2), Tamoxifen (1) | Theoretically based on pharmacology | Moderate | Inhibit CYP3A4 |
| ***Silybum marianum L.Gaertn*** | Milk Thistle | Docetaxel (1), | Vitro | Minor | Maybe inhibit CYP3A4  (more information is needed) |
|  |  | Doxorubicin (1), Ifosfamide (1), Vinblastine (1) |  | Moderate | Maybe inhibit CYP3A4  (more information is needed),  inhibit P-glycoprotein |
| ***Curcuma longa L.*** | Turmeric | Cyclophosphamide (2), Docetaxel* (2), Paclitaxel* (1), Tamoxifen (1) | Vitro | Moderate | Inhibit CYP3A4,  inhibit P-glycoprotein |
|  |  | Docetaxel, Paclitaxel |  | Minor | Inhibit CYP3A4,  Enhances the oral bioavailability of paclitaxel |
| ***Astragalus adscendens boiss**** | Astragalus | Cisplatin (1) | Vitro | Moderate | Stimulate immune function |
| ***Piper nigrum L.*** | Black Piper | Cyclophosphamide (1), Doxorubicin (1), Ifosfamide (1), Etoposide (1), Paclitaxel (1), Vincristine (1) | Vitro | Moderate | Inhibit CYP3A4,  inhibit p-glycoprotein |
| ***Medicago sativa L.*** | Alfalfa | Fluorouracil (1) | Theoretically based on pharmacology | Moderate | increase photosensitivity of drug |
| ***Melilotus officinalis L.*** | Sweet clover | Cyclophosphamide (1), Gemcitabine (1) | Theoretically based on pharmacology | Moderate | Increase hepatotoxicity of the drug |
| ***Hibiscus sabdariffa L.*** | Hibiscus | Doxorubicin (1), Vinblastine (1) | Vitro | Minor | Inhibit CYP3A4 |

*some pairs of herbs and drugs may lead to both potential moderate and minor interactions; therefore, the number of patients for this interaction is mentioned in moderate interactions.
